# Comprehensive study on genetic and chemical diversity of Asian medicinal plants, aimed at sustainable use and standardization of traditional crude drugs

**DOI:** 10.1007/s11418-023-01770-2

**Published:** 2023-12-22

**Authors:** Katsuko Komatsu

**Affiliations:** Institute of Natural Medicine, University of Toyama, 2630 Sugitani, Toyama, 930-0194 Japan

**Keywords:** *Paeonia lactiflora*, *Saposhnikovia divaricata*, *Curcuma*, Molecular identification, Chemical composition, Sustainable use

## Abstract

Our representative studies to achieve sustainable use of crude drugs and ensure their stable quality are introduced: comprehensive studies on genetic, chemical, and sometimes pharmacological diversity of Asian medicinal plants including *Paeonia lactiflora*, *Glycyrrhiza uralensis*, *Ephedra* spp., *Saposhnikovia divaricata*, and *Curcuma* spp., as well as their related crude drugs. (1) For peony root, after genetic and chemical diversity analysis of crude drug samples including white and red peony root in China, the value-added resources with quality similar to red peony root were explored among 61 horticultural *P. lactiflora* varieties, and two varieties were identified. In addition, an optimized post-harvest processing method, which resulted in high contents of the main active components in the produced root, was developed to promote cultivation and production of brand peony root. (2) Alternative resources of glycyrrhiza, ephedra herb and saposhnikovia root and rhizome of Japanese Pharmacopoeia grade were discovered in eastern Mongolia after field investigation and quality assessment comparing Mongolian plants with Chinese crude drugs. Simultaneously, suitable specimens and prospective regions for cultivation were proposed. (3) Because of the wide distribution and morphological similarities of *Curcuma* species, classification of some species is debated, which leads to confusion in the use of *Curcuma* crude drugs. Molecular analyses of the intron length polymorphism (ILP) markers in genes encoding diketide-CoA synthase (DCS) and curcumin synthase (CURS) and *trn*K sequences, combined with essential oils analysis, were demonstrated as useful for standardization of *Curcuma* crude drugs. The above studies, representing various facets, can be applied to other crude drugs.

**Supplementary Information:**

The online version contains supplementary material available at 10.1007/s11418-023-01770-2.

## Introduction

Currently, Japan faces the issue of a super-aging society and consequently aging diseases, syndromes, and conditions such as dementia, sarcopenia, and frailty, and also multifactorial diseases such as life-style-related diseases are increasing. Accordingly, the Japanese Government advocated the National Healthcare Promotion Movement in the twenty-first century [1] with the title of “Health Japan 21,” with aims including extending healthy life expectancy, reducing health disparities, and preventing onset and progression of life-style-related diseases. Japanese traditional medicine (Kampo) has been explored as a potential solution for aging-related diseases, early intervention in pre-symptomatic conditions, and addressing the problem of polypharmacy. Thus, with increased demand for Kampo formulas, their crude drug ingredients must also be supplied. Moreover, sustainable use of crude drugs is fundamentally important in the world. Recently, due to the effects of global climate change and anthropogenic disasters, natural resources have been depleting in many countries, including China. Of the amounts of crude drugs used in Japan, 82.7% are imported from China, and only 10.3% are domestic production [2]. Therefore, medicinal resources of wild and cultivated plants in China must receive close attention. In China, 3000 among 35,000 higher plant species are listed as endangered and 60–70% of endangered plants are used as medicines. Since 2000, the Chinese Government has controlled the collection and export of wild *Ephedra* and *Glycyrrhiza* plants, which are the botanical origins of indispensable crude drugs, ephedra herb and glycyrrhiza, to prevent desertification in northern China [3]. Expansion of cultivation is thought to be the best way to resolve this situation; however, problems such as non-conforming quality of cultivated plants as well as mismatching environmental conditions makes this difficult. Even in China, a half of crude drugs prescribed in the Chinese Pharmacopoeia are derived from wild plants [4].

Here, a strategy for sustainable use of crude drugs is considered as follows: (1) construct a management plan for collection and preservation of medicinal plants at a national level; (2) develop alternative crude drug resources to construct a circulating system of crude drugs in Asia; (3) efficient usage of a crude drug with different botanical origins of the same crude drug name; (4) cultivation of medicinal plants, through the process including proposal for suitable species/strains for cultivation such as value-added medicinal plants, establishing effective and efficient cultivation methods, establishing post-harvest processing and preparation methods, and constructing a comprehensive cycle system from production to consumption.

Concerning the above items, except for the first which is a regulatory concern, our group has conducted comprehensive studies, including field investigation on medicinal plants and traditional medicines, and molecular systematic, chemical, and pharmacological analyses on various crude drugs and their related plants. Clearly, it is necessary to maintain safety and efficacy of crude drugs as well as sustainable uses, and to standardize crude drugs through limiting the acceptable range of diversity for quality assurance purposes. This review introduces some of our studies concerning the above strategies.

### Expanding cultivation in Japan: genetic and chemical diversity of *Paeonia lactiflora* and development of brand peony root

Peony root (Paeoniae Radix) has been widely used as an antispasmodic, analgesic, or astringent in Kampo [5]. It is prescribed as the root of *Paeonia lactiflora* Pallas with no less than 2.0% of paeoniflorin in the Japanese Pharmacopoeia (JP) [6]. Peony root available in the Japanese market (PR, hereafter referring only to crude drugs in the Japanese market) is mainly imported from China and only 2.3% is produced domestically [2]. In China, there are two kinds of peony roots, white peony root (WPR) and red peony root (RPR), which are used for different remedies such as relieving cramps or pains and improving blood stasis or gynecological disease, respectively [5]. The WPR is prescribed as the dried root of *P. lactiflora* which has been boiled and peeled before drying, while RPR is prescribed as the dried root of *P. lactiflora* or *P. veitchii* Lynch in the Chinese Pharmacopoeia (CP) [7]. Most of RPR in the markets is derived from *P. lactiflora*. Therefore, the difference between WPR and RPR with the same botanical origin has been the object of discussion for many years. First, to clarify genetic and chemical differences between the two, the nucleotide sequences of nrDNA internal transcribed spacer (ITS) and contents of eight main constituents were analyzed for specimens of *P. lactiflora*, *P. veitchii*, *P. anomala* Linn., and *P. japonica* Miyabe et Takeda (Table S1), and commercial samples of WPR and RPR available in the Chinese market and those of PR in the Japanese market (Table S2). Second, using the same methods, 81 cultivars cultivated in the Toyama Prefectural Medicinal Plants Center (Table S3) were analyzed and selection performed to produce value-added cultivars to build brand peony root.

#### Genetic diversity of *Paeonia* species and peony root

**Fig. 1:**
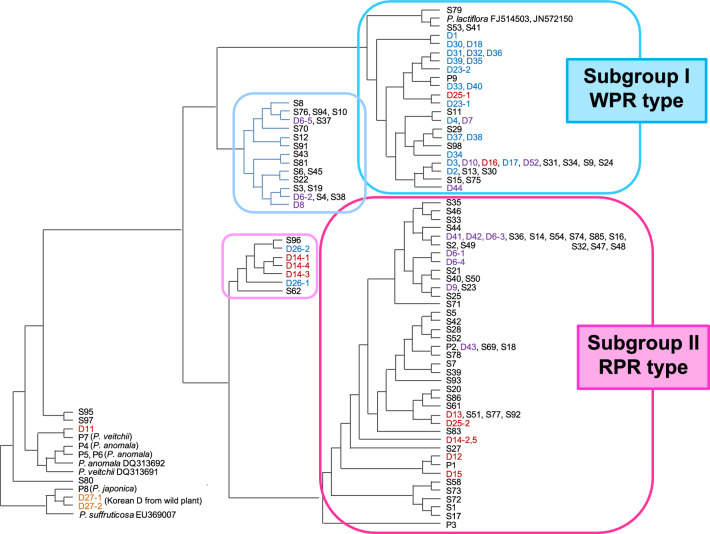
Clustering analysis based on the similarity of ITS sequence. D: Crude drug sample; sky blue in color: Chinese WPR, red color: Chinese RPR, purple color: Japanese PR. P: Plant specimen, S: Cultivar (S31: Kitasaisho, S34: Bonten)

The ITS sequences of specimens of the four *Paeonia* species as well as crude drug samples were of the same length, in which ITS1, 5.8S rRNA gene, and ITS2 regions were 267, 164, and 221 bp, respectively. Almost all the samples of WPR, RPR, and PR were identified as *P. lactiflora*, except for a few RPR samples from Sichuan Province that were identified as *P. veitchii* [8]. Significant intra-species polymorphism of the ITS sequences was detected within *P. lactiflora*. Clustering analysis on the basis of sequence similarity showed that *P. lactiflora* formed a large group distinct from *P. veitchii*, *P. anomala*, *P. japonica*, and *P. suffruticosa* Andrews (Fig. 1). Within the *P. lactiflora* group, there were two main subgroups: I and II. The *P. lactiflora* plants cultivated in southern China and Chinese WPR produced in Anhui, Zhejiang, and Sichuan provinces, as well as Japanese PR including Japanese medicinal cultivars “Bonten (S34)” and “Kitasaisho (S31),” belonged to subgroup I (WPR-type); whereas, *P. lactiflora* plants growing wild in northern China (P1, P2) and Mongolia (P3), and Chinese RPR (D12-D15) fell into subgroup II (RPR-type). Most horticultural varieties from Japan belonged to subgroup II.

The ITS sequences deposited in the International Nucleotide Sequence Database included two main types, differing by three nucleotides at positions 69, 458, and 523: type 1 showed three cytosines (C–C–C; U27682) [9], while type 2 had thymine (T), adenine (A), and T (T–A–T; JN572150) [10] at the three sites. According to the nucleotides at these three sites, crude drug samples and plants were divided into two main groups corresponding to the two subgroups (Fig. 1). One group showed T_69_-A_458_-T_523_ at the three sites, which was the same as for type 2. The other group showed additive nucleotides (double nucleotides detected at the same site) of Y (T & C), M (A & C), and Y at the three sites (Y_69_-M_458_-Y_523_), respectively. The two Japanese medicinal cultivars showed a sequence identical to a type of sequence from WPR (AB920144) of subgroup I. The five PR samples produced in Nara Prefecture (D7, D9, D10, D42, and D44), usually called “Yamato Shakuyaku,” were divided into two subgroups: I and II. Moreover, the PR from Niigata Prefecture (D6) was a mixture of individuals belonging to two subgroups, indicating Japanese PR was not only derived from medicinal cultivars of *P. lactiflora*.

#### Chemical diversity of *Paeonia* species and peony root

**Fig. 2:**
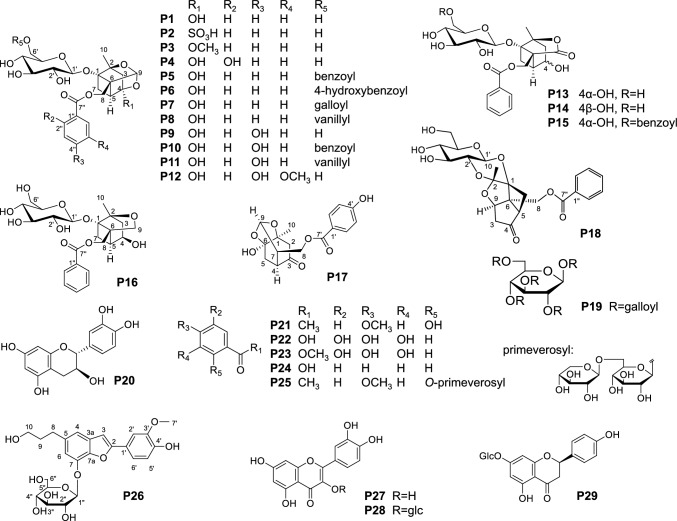
Structures of reference standard compounds. **P1**, paeoniflorin; **P2**, paeoniflorin sulfonate; **P3**, 4-*O*-methylpaeoniflorin; **P4**, salicylpaeoniflorin; **P5**, benzoylpaeoniflorin; **P6**, mudanpioside C; **P7**, galloylpaeoniflorin; **P8**, mudanpioside J; **P9**, oxypaeoniflorin; **P10**, benzoyloxypaeoniflorin; **P11**, 6’-*O*-vanillyloxypaeoniflorin; **P12**, mudanpioside E; **P13**, albiflorin; **P14**, 4-*epi*-albiflorin; **P15**, paeonivayin; **P16**, paeoniflorol; **P17**, 4’-hydroxypaeoniflorigenone; **P18**, lactiflorin; **P19**, 1,2,3,4,6-penta-*O*-galloyl-β-d-glucose; **P20**, (+)-catechin; **P21**, paeonol; **P22**, gallic acid; **P23**, methyl gallate; **P24**, benzoic acid; **P25**, paeonolide; **P26**, paeonibenzofuran; **P27**, quercetin; **P28**, quercetin-3-*O*-β-d-glucopyranoside; **P29**, (*2R*)-(-)-naringenin-7-*O*-β-d-glucopyranoside

**Fig. 3:**
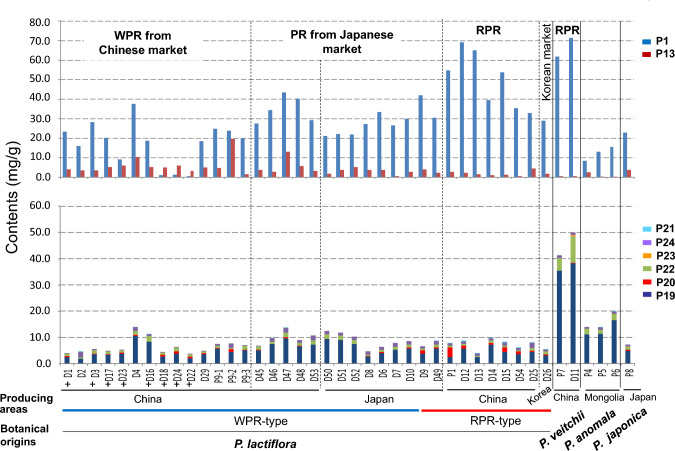
Contents of 8 components in the different types of peony root and the roots of four related species including *P. lactiflora*, *P. veitchii*, *P. anomala* and *P. japonica*. D: crude drug sample; ^+^: samples processed by sulfur-fumigation. P: plant specimens; P9, P1 and P7 were collected in Zhejiang, Inner Mongolia and Sichuan, respectively. Contents of **P1** and **P13** are shown in upper part and those of **P19**-**P24** are shown in lower part

The contents of eight components—paeoniflorin (**P1**), albiflorin (**P13**), 1,2,3,4,6-penta-*O*-galloyl-β-d-glucose (**P19**), (+)-catechin (**P20**), paeonol (**P21**), gallic acid (**P22**), methylgallate (**P23**), and benzoic acid (**P24**)—were quantitatively analyzed to clarify chemical properties of *P. lactiflora*, *P. veitchii*, *P. anomala*, and the crude drug samples including WPR, PR, and RPR (Fig. 2). Within the commercial samples derived from *P. lactiflora*, RPR samples produced in northern China had obviously higher contents of **P1**, **P20**, and **P21** but lower content of **P13** compared to WPR produced in southern China and most PR produced in Japan [8] (Fig. 3). Among 11 WPR samples, eight contained less than the 2.0% of paeoniflorin specified in the JP. Comparing high-performance liquid chromatography (HPLC) chromatograms of these samples with those of the PR collected from Japanese markets, commonly showed a conspicuous peak at retention time around 10.4 min. Further analysis using liquid chromatography (LC)/mass spectrometry (MS) clearly indicated that this notable peak was paeoniflorin sulfonate (**P2**), which could be produced by traditional processing with sulfur fumigation [11]. This indicated that the WPR available in Chinese markets was usually processed by sulfur fumigating, resulting in an extremely low **P1** content; whereas the PR available in the Japanese market was not treated with this process. Apart from the WPR samples in which **P2** was detected, quantitative data of the six constituents excluding **P23** and **P24** in *Paeonia* specimens and commercial samples was subjected to principal component analysis (PCA) and the PCA score plot showed four separate clusters of all samples. Besides the respective groups of *P. veitchii* and *P. anomala*, samples derived from *P. lactiflora* were clearly classified into two groups: one group included RPR (RPR group) and the other group was composed of WPR, PR produced in China, and most PR produced in Japan (WPR/PR group). The former was characterized by high **P21**, **P20**, and **P1** contents, and the latter by a relatively high content of **P13**. Moreover, the *P. veitchii* group was far from the other groups and had high contents of **P1**, **P19**, and **P22**. The grouping in the PCA score plot was in accordance with clustering based on the similarity of ITS sequences. This result indicated that WPR and RPR were not only geographically isolated, but also genetically and chemically separated.

**Fig. 4:**
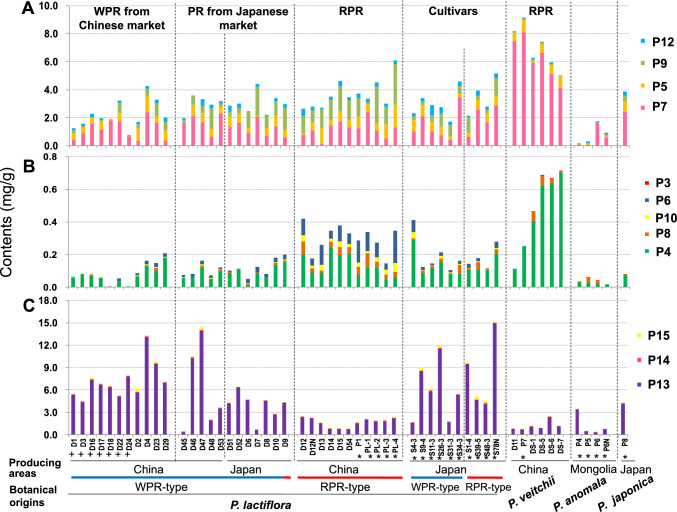
Contents of 11 monoterpenoids in the different types of peony root and the roots of four related species including *P. lactiflora*, *P. veitchii*, *P. anomala* and *P. japonica*. **A** and** B**: Contents of peoniflorin-type monoterpenoids, **P5**, **P7**, **P9** and **P12** in **A**, and **P3**, **P4**, **P6**, **P8** and **P10** in **B**;** C**: Contents of albiflorin-type monoterpenoids, **P13**–**P15**. *: plant specimens; ^+^: samples processed by sulfur-fumigation

In addition, monoterpenoid profiling was performed using LC coupled with ion trap and time-of-flight MS (LC–IT-TOF-MS) to precisely characterize and quantify different types of peony root and the roots of related *Paeonia* species. The MS/MS fragmentation patterns of monoterpenoids with paeoniflorin (PF)-, albiflorin (AF)-, and sulfonated paeoniflorin (PFS)-type of skeletons were elucidated, which provided basic clues enabling subsequent identification of 35 monoterpenoids in LC–MS profiles of *Paeonia* species (Fig. 2). Mudanpioside C (**P6**) was the characteristic component of *P. lactiflora*, and 4-*O*-methyl-paeoniflorin (**P3**) was only detected in *P. veitchii* and *P. anomala*. Notably, six PFS-type monoterpenoids were detected in sulfur-fumigated WPR samples [12]. Quantification of 15 main monoterpenoids including 10 PF-type (**P1**, **P3–P10**, and **P12)**, three AF-type (**P13–P15**), and two other types, a new compound paeoniflorol (**P16**) and lactiflorin (**P18**), in 56 samples revealed that five monoterpenoids [**P1**, benzoylpaeoniflorin (**P5**), galloylpaeoniflorin (**P7**), oxypaoniflorin (**P9**), and **P13**] predominated in all samples, but with quite different relative contents. Of the samples derived from *P. lactiflora*, RPR samples showed higher contents of five PF-type monoterpenoids [salicylpaeoniflorin (**P4**), **P6**, mudanpioside J (**P8**), **P9**, and benzoyloxypaeoniflorin (**P10**)] as well as **P1** compared to WPR/PR, but comparatively lower contents of AF-type monoterpenoids [a new compound, 4-*epi*-albiflorin (**P14**), paeonivayin (**P15**), and **P13**] (Fig. 4). For the two other types of monoterpenoids, **P16** had a high content in RPR, whereas **P18** had a high content in WPR and PR. The above differences in monoterpenoid profiles between WPR and RPR might be some of the reasons for their different traditional uses and different bioactivities including anti-allergic effects. The samples derived from *P. veitchii* had the highest **P4** and **P7** contents, very low **P9** and **P10** contents, and no **P6**, and clearly differed from those derived from other sources [12].

The same analytical methods as peony root were applied to two medicinal and 61 horticultural cultivars of *P. lactiflora*, which were genetically of RPR- and WPR-types. There were high similarities between RPR- and WPR-types of cultivars in regard to the eight main constituents (Fig. S1) [13]. Moreover, monoterpenoid composition of the respective *P. lactiflora* cultivars showed high similarity regardless of RPR- or WPR-type (Fig, 4) [12], differing from the situation between commercial WPR and RPR.

#### Anti-allergic activity of peony root and *P. lactiflora* horticultural cultivars and their active compounds

Because several studies have reported anti-allergic activity of peony root [14], a bioactivity screening experiment concerning the inhibitory effect against immunoglobulin E (IgE)-mediated mast cell degranulation was performed to compare the activity of RPR from the Inner Mongolia Autonomous Region of China and PR from Japan and to determine a characteristic cultivar with anti-allergic activity among 17 horticultural cultivars. Among them, a RPR sample and two cultivars, “Edulis Superba (ES)” and “Harunoyosooi (HY),” significantly inhibited *β-*hexosaminidase release stimulated by 2,4-dinitrophenylated bovine serum albumin on IgE-sensitized rat basophil leukemia-2H3 cells at a concentration of 1.0 mg/mL. In order to elucidate the active compounds, bioassay-guided fractionations were subsequently conducted on the RPR sample and cultivar ES, and anti-allergic activities of isolated compounds were, respectively, examined. From the 60% and 80% aqueous methanol subfractions of methanol extract of RPR sample that showed activity, 29 compounds were isolated and identified as three new monoterpenoids [**P14**, **P16**, and 4′-hydroxypaeoniflorigenone (**P17**)], 14 known monoterpenoids, five flavonoids, and seven other types of compounds [15]. In the same way, 26 compounds were isolated from the ethyl acetate- and *n*-butanol-soluble fractions of methanol extract of cultivar ES, including one new norneolignan, paeonibenzofuran (**26**), five monoterpenoids, 10 flavonoids, and 10 other types of compounds [16]. Among compounds isolated from the RPR sample, nine monoterpenoids (**P1**, **P4**, **P5**, **P7**, **P8**, **P10**, **P11**, **P16**, and **P18**), as well as **P19** and **P23**, showed moderate anti-allergic activities (Table S4). Among monoterpenoids, a new compound **P16** exhibited the greatest effectiveness (IC_50_ 41.17 μM), followed by **P4** and **P7**. However, AF-type monoterpenoids had no activity [15]. Moreover, mudanpioside E (**P12**), quercetin (**P27**), and quercetin-3-*O*-β-d-glucopyranoside (**P28**) isolated from cultivar ES, showed potent inhibitory activity (IC_50_ 40.34, 25.05, and 42.55 μM, respectively), followed by paeonolide (**P25**), (*2R*)-(-)-naringenin-7-*O*-β-d-glucopyranoside (**P29**), and **P26** [16]. Based on these experimental results, two horticultural cultivars with moderate anti-allergic activity were selected as promising candidates with potential as medicinal resources of RPR, as well as the active components being clarified.

#### Development of brand peony root in Toyama―selection from horticultural cultivars and establishment of appropriate post-harvest processing methods

A medicinal cultivar “Bonten (BT)” belonging to WPR-type has been widely cultivated in Toyama Prefecture. To find new resources of peony root of RPR-type from *P. lactiflora* cultivars, with both horticultural and medicinal utilities, PCA was performed on quantitative data of six constituents (**P1**, **P13**, and **P19–P22**) from 63 *P. lactiflora* cultivars added to those from commercial samples except **P2**-containing samples, and *Paeonia* specimens. In the PCA score plot, samples and specimens derived from *P. lactiflora* were clearly classified into two groups: RPR and WPR/PR [13]. Three horticultural cultivars were included in the RPR group; the two cultivars ES and HY among them showed anti-allergic activity as described in the previous section, and were nominated as candidates for brand peony roots.

**Fig. 5:**
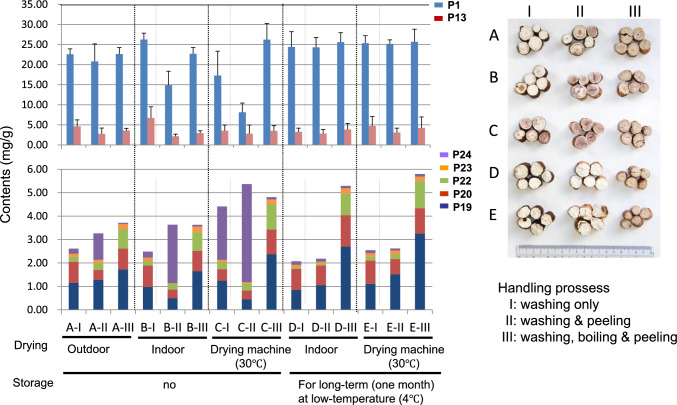
Contents of 7 components in the roots produced by 15 kinds of post-harvest processing methods. Average contents and S.D. of **P1** and **P13** are shown in upper part and average contents of **P19**, **P20**, **P22–P24** are shown in lower part (*n* = 5). Photo of cross-sections of the roots treated with different processing methods is shown

Next, with the aim to determine the influence of different post-harvest processing methods of boiling, peeling, drying, and storing on chemical composition and morphological features of the produced peony root and to establish an appropriate and practicable method for production of brand peony root with superior quality, 15 kinds of processing methods were applied to the 15 groups of fresh roots of cultivar BT after 4 years of cultivation. The roots produced were analyzed using HPLC and the contents of eight components (**P1**, **P13**, and **P19–P24**) and internal root color were compared (Fig. 5). The results showed that low temperature (4 °C) storage of fresh roots for approximately 1 month after harvest resulted in stable and high content of **P1**, possibly due to suppression of enzymatic degradation including enzymes involved in paeoniflorin hydrolysis [17]. This storage also prevented discoloration of roots and facilitated production of desirable bright color in roots, traditionally believed to be of high quality. In addition, quantitative ^1^H nuclear magnetic resonance (qHNMR) analysis demonstrated that sucrose content increased significantly in root after low-temperature storage for 1 month, resulting in increased ethanol extract yield [18]. Boiling treatment triggered decomposition of polygalloylglucoses and led to significantly increased contents of **P19**, **P22**, and **P23**, shown by monitoring gallotannin changes using LC-IT-TOF–MS. Peeling treatment resulted in decreased **P13** content; additionally in the group without boiling, the **P20** content also decreased. For roots washed and boiled after low-temperature storage, **P19** content was slightly higher for no peeling compared to peeling treatment, and for drying at 30 °C using a drying machine compared with indoor drying. As a result, the optimized processing method to produce high contents of main active components in the root was low-temperature storage for approximately 1 month after harvest, followed by washing, boiling, and drying at 30 °C with a drying machine [17]. Similar experiments to those described above were applied to two *P. lactiflora* cultivars: ES and HY. Following application of the optimized processing method, the main constituents were quantified and compared among three cultivars, with the order for levels of **P1** and **P13** of ES > HY > BT, and for **P20** of HY > ES > BT. Moreover, **P23** was found in cultivars ES and BT. Although **P21** was not detected following the optimized processing roots of the three cultivars, ES and HY contained **P21** in the dried roots treated without boiling. Using the above results, we selected suitable cultivars and processing methods. Recognizing the potential of these discoveries, a collaborative project between academia and local government is currently in progress in Toyama Prefecture to produce brand peony root.

### Development of alternative crude drug resources: quality assessment of plant resources of glycyrrhiza, ephedra herb, and saposhnikovia root and rhizome from Mongolia

Around 3000 species of vascular plants are distributed in Mongolia: 812 species are efficacious medicinal plants, and 200 species have been used as formulaic ingredients in Mongolian traditional medicine [19]. Mongolian medicinal plants are also attractive as sources of crude drugs used in traditional Chinese medicine and Kampo, such as *Glycyrrhiza uralensis* Fischer, *Ephedra sinica* Stapf, *E. equisetina* Bunge, and *Saposhnikovia divaricata* (Turcz.) Schischk. Qualities of these plants were assessed in a collaborative study with Mongolian researchers to inform an ongoing conservation program for the efficient usage of medicinal plants by the Mongolian Government.

#### Resources of glycyrrhiza (Glycyrrhizae Radix)

For glycyrrhiza, one of the crude drugs with export restricted by the Chinese Government, to reveal chemical characteristics of *G. uralensis* growing in Mongolia, eight major bioactive constituents in the underground parts were quantitatively analyzed and compared with glycyrrhiza produced in China [20]. Most of the 15 specimens from eastern, southern, and western Mongolia contained 26.95–58.55 mg/g of glycyrrhizin, exceeding the criterion assigned in the JP [6], and was highest in the sample from Tamsagiyn Hooloy, Dornod Province, eastern Mongolia. The total content of three flavanones (liquiritin apioside, liquiritin, and liquiritigenin) was in the range of 3.00–26.35 mg/g and of three chalcones (isoliquiritin apioside, isoliquiritin, and isoliquiritigenin) was 1.13–10.50 mg/g. The content of glycyrrhizin and subtotal contents of flavanones and chalcones from Mongolian *G. uralensis* were obviously lower than those of the crude drugs available in the Japanese market derived from Chinese wild specimens, but higher than those derived from cultivated specimens in the Chinese market. Glycycoumarin, a constituent specific to *G. uralensis*, was detected in all Mongolian specimens and its content was higher in samples from Sergelen and Tamsagiyn Hooloy, which were comparable to that of Tohoku-kanzo in Japan derived from wild Chinese *G. uralensis*. Thus, Mongolian *G. uralensis* could be a glycyrrhiza resource and was generally of JP grade. However, the producing area should be taken into consideration for ensuring high quality.

#### Resources of ephedra herb (Ephedrae Herba)

After a field survey of *Ephedra* plants in Mongolia, molecular and chemical assessments on plant specimens were conducted to clarify whether they could be an alternative resource of ephedra herb used in Japan [21]. The distribution of *E. sinica*, *E. equisetina*, *E. monosperma* Gmelin ex C. A. Meyer, *E. przewalskii* Stapf, *E. glauca* Regel, *E. regeliana* Florin, *E. lomatolepis* Schrenk, and unknown *Ephedra* sp. (assumed to be *E. dahurica* Turz.) was confirmed. Among them, *E. sinica* and *E. equisetina* are prescribed as the botanical origin of ephedra herb in JP [6]. On the basis of nucleotide sequences of nuclear 18S rRNA gene and chloroplast *trn*K gene, *E. sinica*, *E. equisetina*, and *E. monosperma* presented completely identical sequences to the corresponding species from China. Since the population in southwestern Mongolia showed a high likelihood of hybrid origin, further sequence analysis of the partial nuclear ITS1 region was conducted to obtain detailed evidence of hybridization status as well as to elucidate the marker sequences of pure lines of each species [22]. As a result, the ITS1 sequences from all eight *Ephedra* species were roughly divided into five types: I–V. *E. equisetina* and *E. monosperma* had similar sequences (Type V), differing from other species. Although the remaining five species possessed similar sequences, they were divided into four types based on the nucleotides at four informative sites. Among them, type II sequences had additive nucleotides at four sites observed in *E. sinica*, *Ephedra* sp., *E. glauca*, and *E. regeliana*, which provided useful information for tracing hybrid origin. Morphological, genetic and distribution evidences suggested that hybridization of *Ephedra* species occurred widely in southwestern Mongolia, and several *Ephedra* species including *E. przewalkskii* were involved in these events. Integrated with *trn*K-, 18S-, and ITS-sequence types, pure lines of each species were proposed. The pure line (type I [AB600683]) of *E. sinica* was only found in eastern areas, while *E. equisetina*, which consisted of pure line, was found in the mountainous region of central–western areas. Quantitative analysis of five ephedrine alkaloids [ephedrine (**E1**), pseudoephedrine (**E2**), norephedrine, norpseudoephedrine, and methylephedrine] revealed that almost all Mongolian *Ephedra* plants, except *E. przewalskii* and *E. lomatolepis*, contained high amounts of total ephedrine alkaloids (TAs), with range 1.86–4.90% of dry weight [21]. The *E. sinica* (types I and II) contained 1.95–4.16% TAs and showed a higher percentage of **E1** in TAs than other species. Within *E. sinica*, types I and II specimens collected from eastern grassland areas showed a high proportion of **E1** in TAs (mean 51.4% and 54.0%, respectively); whereas, type II specimens from central areas showed a high proportion of **E2** in TAs (mean 55.5%). The proportions of **E1** and **E2** might be affected more by growing environment than genetic factors. *Ephedra equisetina* had the highest content of TAs (3.98–4.90%), with more than 90% of TAs being **E2**. Both *E. sinica* and *E. equisetina* satisfied the JP criterion [6] of containing no less than 0.7% of summed content of **E1** and **E2**, with 1.43–3.68% and 3.81–4.59%, respectively. Therefore, these two species growing in Mongolia were a suitable new resource of ephedra herb used in Japan. Given that the pure line of *E. sinica* is limited to eastern areas, and *E. equisetina* has a limited distribution, promoting the cultivation of selected specimens in suitable regions is crucial for ensuring a stable supply of ephedra herb and supporting environmental preservation.

#### Resources of saposhnikovia root and rhizome (Saposhnikoviae Radix)

Saposhnikovia root and rhizome (SR) derived from *Saposhnikovia divaricata* (Turcz.) Schischk. have been used as a diaphoretic, febrifuge, analgesic, and anti-phlogistic in China and Japan, including frequently as an ingredient in Kampo formulas [5]. In recent years, natural SR resources have been depleted because of increasing demand, therefore, a large amount of SR derived from cultivated plants has become available in the Chinese and Japanese markets. However, some of these do not meet the CP and JP requirements due to lower amounts of prim-*O*-glucosylcimifugin (**S1**) and 4′-*O*-β-d-glucosyl-5-*O*-methylvisamminol (**S3**) and the higher yield of dilute-ethanol-soluble extract [6, 7]. For sustainable utilization of SR resources through high-performance cultivation by selecting suitable plant resources and cultivation areas, Mongolia was chosen because of the large population of wild growing *S. divaricata*, especially in the eastern part including Khentii and Dornod Provinces, and field investigation and metabolomic analysis were performed on collected plant specimens.

**Fig. 6:**
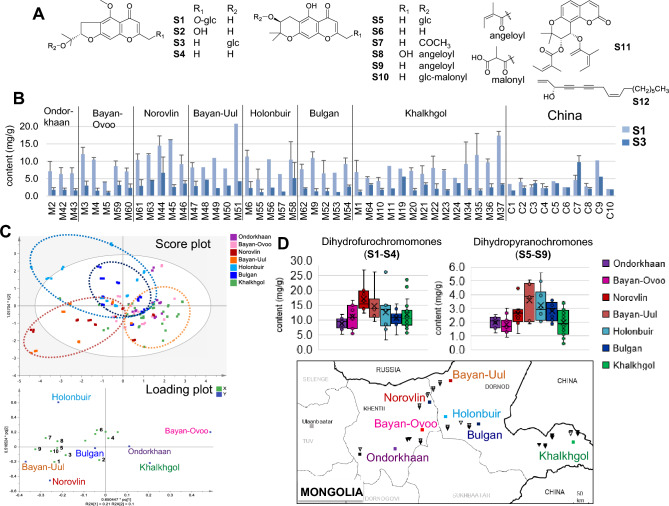
Quantification of metabolites in *Saposhnikovia divaricata* roots from Mongolia by HPLC–DAD. **A** Structures of reference standard compounds; **B** Average contents and S.D. of **S1** and **S3** in Mongolian specimens and saposhnikovia root and rhizome from China; **C** OPLS-DA of Mongolian *S. divaricata* specimens based on HPLC data; **D** Box plot of the levels of total dihydrofurochromones (**S1**–**S4**) and total dihydropyranochromones (**S5**–**S9**) in Mongolian specimens. **S1**, prim-*O*-glucosylcimifugin; **S2**, cimifugin; **S3**, 4′-*O*-β-d-glucosyl-5-*O*-methylvisamminol; **S4**, 5-*O*-methylvisamminol; **S5**, sec-*O*-glucosylhamaudol; **S6**, hamaudol; **S7**, 3′-*O*-acetylhamaudol; **S8**, ledebouriellol; **S9**, 3′-*O*-angeloylhamaudol; **S10**, 3’-*O*-(6’’-*O*-malonyl)-glucosylhamaudol; **S11**, praeruptorin B; **S12**, panaxynol

To evaluate the quality of Mongolian *S. divaricata*, metabolomic profiling of all root parts of 43 specimens from Mongolia, as well as eight SR samples and two plant specimens from China, were conducted using LC-IT-TOF–MS. The LC–MS profiles of the 70% methanol extracts of the specimens and SR samples showed uniformity, and 13 chromones and 17 coumarins were tentatively identified [23]. Among them, a new compound, 3′-*O*-(6″-*O*-malonyl)-glucosylhamaudol (**S10**) [24] and 17 known compounds were isolated and unambiguously identified. Orthogonal partial least squares-discriminant analysis (OPLS-DA) based on LC–MS data revealed that Mongolian specimens were clearly distinguished from Chinese SR and characterized by an abundance of **S1**. Moreover, Mongolian specimens could be discriminated by their growing regions based on the content of eight compounds (**S1–S3**,** S6**, **S7**, and **S9–S11**) (Fig. 6A). Specimens from Khalkhgol in far eastern Mongolia contained higher amounts of dihydrofurochromones **S1**, cimifugin (**S2**), and **S3**, while those from Holonbuir contained relatively higher amounts of hamaudol (**S6**), 3′-*O*-acetylhamaudol (**S7**), 3′-*O*-angeloylhamaudol (**S9**), and praeruptorin B (**S11**) [23].

Subsequently, to accurately determine the contents of metabolites in *S. divaricata* roots from eight different regions of Mongolia and to investigate their geographical variation, nine chromones (**S1–S9**) and four coumarins were quantified using HPLC-diode-array detection. All 44 Mongolian specimens contained **S1** (3.98–20.79 mg/g) and **S3** (1.06–6.68 mg/g), with their total content (5.04–25.06 mg/g) exceeding the criterion (2.4 mg/g) assigned in the CP [24] (Fig. 6B). The contents of **S1**, **S7**, ledebouriellol (**S8**), and **S9** were significantly higher in the Mongolian specimens than in Chinese SR samples. Moreover, specimens from Norovlin in northeastern Mongolia had the highest level of total dihydrofurochromones (**S1–S4**; 12.20–26.80 mg/g) including **S1** (9.18–16.22 mg/g) and **S3** (2.60–6.68 mg/g), while the level of dihydrofurochromones was more variable in specimens from Khalkhgol (5.59–23.41 mg/g) and Holonbuir (3.37–26.12 mg/g) (Fig. 6D). The level of total dihydropyranochromones (**S5–S9**) tended to be higher in specimens from Bayan-Uul (1.86–5.04 mg/g) in northeastern Mongolia. The OPLS-DA based on HPLC data revealed that the Mongolian specimens tended to be separated into three groups based on growing regions, in which several chromones contributed to each group (Fig. 6C). Furthermore, for characterizing metabolites such as polyacetylenes and sugars, qHNMR analysis showed that Mongolian specimens had less sucrose and a substantial amount of polyacetylenes including panaxynol (**S12**) [24]. Thus, the chemical characteristics of Mongolian *S. divaricata* specimens were clarified and showed that specimens from northeast Mongolia, including Norovlin, had superior properties due to higher amounts of major chromones. Although Norovlin was proposed as a prospective region for *S. divaricata* cultivation, the amount of the compounds varied widely due to growing conditions. Therefore, cultivation method should be studied as a next step.

### Standardization of crude drugs: genetic polymorphism and essential oil composition of Asian *Curcuma* species and crude drugs

Genus *Curcuma* comprises approximately 120 species, and rhizomes of approximately 30 species have been used to treat stomach disorder and the syndrome of blood stasis in traditional medicines, as well as for spices, dyes, and cosmetics. Recently, with the increasing popularity of foods with health claims and so-called “health food” in Japan and other countries, *Curcuma* rhizomes are frequently used worldwide, such as turmeric called “Jianghuang” in Chinese, “Ukon” in Japanese, and “Haldi” in Hindi derived from *C. longa* Linn.; “Temu lawak” in Javanese derived from *C. zanthorrhiza* Roxb.; “Ezhu” in Chinese derived from *C. phaeocaulis* Valeton, *C. kwangsiensis* S. G. Lee et C. F. Liang, or *C. wenyujin* Y. H. Chen et C. Ling; and “Gajutsu” and “Haruukon” in Japanese derived from *C. zedoaria* (Christm.) Roscoe and *C. aromatica* Salisb., respectively, cultivated in Japan. However, because of the wide distribution and morphological similarities of *Curcuma* species, classification of some species is debated and nomenclature is inconsistent among countries, especially for *C. zedoaria* and *C. aromatica* [25]. This situation leads to confusion in the use of *Curcuma* crude drugs and affects their safe and efficient use. Such crude drugs with wide distribution have a more critical problem with standardization than with sustainable use. Thus, aiming at standardization of *Curcuma* crude drugs, a study on genetic and chemical diversity of Chinese and Japanese *Curcuma* species and their derivative drugs was carried out and the scope was expanded into Asian *Curcuma*.

#### Genetic polymorphism of Chinese and Japanese *Curcuma* species and botanical origin of *Curcuma* crude drugs

##### 18S rRNA gene and *trnK* gene sequences

Five *Curcuma* species, *C. longa*, *C. phaeocaulis*, *C. kwangsiensis*, *C. zedoaria*, and *C. wenyujin* described as the botanical origins of Jianghuang/Ukon, Ezhu/Gajutsu, and Pian-Jianghuang in CP and JP, and *C. aromatica* used as health foods in Japan were obtained from field investigation. The nuclear 18S rRNA (biparental inheritance) and chloroplast *trn*K gene (maternal inheritance) sequences of specimens of each species were determined [26, 27]. The 18S rRNA gene sequences of six species were 1810 bp in length and highly conserved. Only one site difference was found at nucleotide position 234, with thymine (T) in *C. kwangsiensis* and *C. zedoaria* from Japan [*C. zedoaria* (Jp)] and cytosine (C) in four other species. The *trn*K gene length ranged within 2698–2705 bp depending on the species and had nine base substitutions and three indels in the intron region. Comparing the sequences of five species, except for *C. aromatica*, four base substitutions were observed. The poly-T numbers from nucleotide position 502 was in the range of 10–14, according to species or specimens. The sequence of *C. phaeocaulis* possessed a 4-bp insertion repeat at nucleotide position 730. In *C. aromatica* from Japan [*C. aromatica* (Jp)] a 8-bp deletion, a 14-bp insertion repeat, and five base substitutions were observed. The *C. kwangsiensis* specimens were divided into two groups on the basis of nucleotide differences at four positions [27, 28]. Morphologically, one group had leaf blades with a purple-colored band along the midrib and lateral spikes, whereas another group had pubescent leaf blades without a purple band and central spikes—these we assigned as pl and gl types, respectively. The sequences of pl and gl types of *C. kwangsiensis* were identical to those of *C. zedoaria* (Jp) and *C. wenyujin*, respectively. A series of sequence analysis suggested that six *Curcuma* species distributed in China and Japan could be converged in five types: Ltk [AB047738], Ptk [AB047735], Atk [AB047731], K(pl)Ztk [AB047744], and K(gl)Wtk [AB047745]. All *C. kwangsiensis* specimens and a few specimens of unknown species collected in cultivated fields of Guangxi Zhuangzu Autonomous Region had the K(gl)Wtk type of sequence, although the 18S rRNA gene sequence at position 234 suggested they were heterozygote, and essential oil (EO) compositions of the rhizome varied [29].

With regard to the identification of *Curcuma* crude drug samples, their *trn*K gene sequences were determined after three divided regions were amplified using a nested PCR method. Jianghuang/Ukon samples produced in China available in Chinese and Japanese markets possessed the Ltk type and Gajutsu samples produced in Kagoshima Prefecture, Japan, had the K(pl)Ztk type of sequence, suggesting origins were *C. longa* and *C. zedoaria* (Jp), respectively. Pian-jianghuang samples produced in Zhejiang Province possessed the K(gl)Wtk type of sequence, which might be *C. wenyujin* judging from the production area. Among Ezhu/Gajutsu samples produced in China available in Japanese markets, those from Sichuan Province possessed the Ptk type of sequence, suggesting *C. phaeocaulis*. On the one hand, those from southern China, mostly Guanxi, were mixtures of individuals with K(gl)Wtk, K(pl)Ztk, or Ptk type of sequence. Moreover, there were individuals detected with one or two base substitutions, frequently at nucleotide position 2582 in these three types of sequences. On the other hand, to investigate the nucleotide difference at position 234 in 18S rRNA sequence of these individuals, PCR–RFLP analysis using restriction enzyme *Ban* II was performed, which recognizes the sequence GRGCY/C and digests the PCR product with C at this position. As a result, the PCR products from most individuals gave both not digested band and digested two bands in electrophoretograms, suggesting heterozygote Y (T & C). In addition, EO components of Ezhu/Gajutsu samples were investigated. The compositions of both samples from Sichuan Province and from Japan were similar to each other, mainly containing curzerenone, furanodienone, and curcumenol; whereas compositions of samples from Guanxi Zhuangzu Autonomous Region including individuals with genetic polymorphism varied [30]. Thus, Ezhu/Gajutsu samples from Guangxi lacked genetic and chemical stability.

#### Intron length polymorphism markers in genes encoding diketide-CoA synthase and curcumin synthase

We investigated a new method for discriminating *Curcuma* species and for standardizing crude drugs, especially Ezhu/Gajutsu, using molecular analysis based on the intron length polymorphisms (ILPs) in genes encoding diketide-CoA synthase and curcumin synthase, because *trn*K sequence comparison could not differentiate *C. kwangsiensis* (gl type) from *C. wenyujin* with the same K(gl)Wtk type of sequence. Curcuminoids are the most important components in *Curcuma* drugs. The curcuminoid biosynthesis route of *C. longa* has been determined, in which two type III polyketide synthases, diketide-CoA synthase (DCS) and curcumin synthase (CURS1) are involved. The DCS catalyzes formation of feruloyldiketide-CoA by condensing feruloyl-CoA and malonyl-CoA; and CURS1 mainly catalyzes formation of curcumin from feruloyl-CoA and the feruloyldiketide-CoA produced by the action of DCS [31]. Moreover, CURS2 and CURS3, isozymes of CURS1, are also responsible for curcuminoid synthesis and have been discovered in *C. longa*. The CURS2 prefers feruloyl-CoA as a starter substrate and CURS3 prefers both feruloyl-CoA and *p*-coumaroyl-CoA [32]. Similarly, DCS2, another isozyme of DCS (DCS1), has been identified. Two isozymes of DCS similar to DCS1 and DCS2 and three isozymes of CURS similar to CURS1, CURS2, and CURS3 of *C. longa* in amino acid sequence were obtained from several *Curcuma* species irrespective of whether they contain curcuminoids in their rhizomes. The nucleotide sequences of *DCS1* and *DCS2* of *C. longa*, which encode DCS1 and DCS2, respectively, contain two introns (*DCS* intron I and II), which divide the coding region into three exons; and those of *CURS1*, *CURS2*, and *CURS3*, which encode CURS1, CURS2, and CURS3, respectively, each contain two exons and one intron (*CURS* intron). Although the sequences of exons were conserved, the sequences of introns were quite variable, even in size, which offer potential for diversity analysis. The ILPs are useful genetic markers because they represent variation in the transcribed portion of the genome. The ILP markers developed in various gene regions of many plant species are used for gene tagging, diversity analysis, and comparative studies [33, 34]. Therefore, we developed a molecular method using ILP markers in *DCS1*, *DCS2*, and *CURS1–CURS3* to discriminate *Curcuma* species and identify their related crude drug samples [35].

First, two intron regions I and II in *DCS1* and *DCS2* and one intron region in *CURS1–CURS3* were amplified separately via PCR using each of the three pairs of primers. One primer of each pair was labeled with different fluorescent dyes, enabling the respective amplicons to be detected and discriminated. The PCR product of each intron region was mixed with size standard and analyzed using capillary electrophoresis. The length of the amplified fragments was then determined by comparison with size standard markers that included 36 single-stranded labeled fragments. As the result, specimens of *C. phaeocaulis*, *C. zedoaria* (Jp), *C. wenyujin*, and *C. aromatica* (Jp) showed a species-specific fragment profile, whereas each specimen of *C. kwangsiensis* (gl type) had an individual fragment profile, showing intraspecies variation. However, all *C. kwangsiensis* (gl type) specimens fell into a single group in the dendrograms constructed by the fragment patterns in the three intron regions of each specimen and commercial sample [35]. Thus, *C. kwangsiensis* (gl type) specimens were distinguishable from *C. wenyujin* specimens using the ILP profile. Combined with variable chemical constituents in *C. kwangsiensis* specimens, we speculated that there was cross-hybridization between *C. kwangsiensis* (gl type) and other *Curcuma* species, supported by the fact that seed setting sometimes occurs in this species. The molecular method we developed has potential for global classification of *Curcuma*.

#### Molecular analysis of Asian *Curcuma* species and related crude drugs based on ILP markers in *DCS* and *CURS* genes and *trnK* gene sequences

**Fig. 7:**
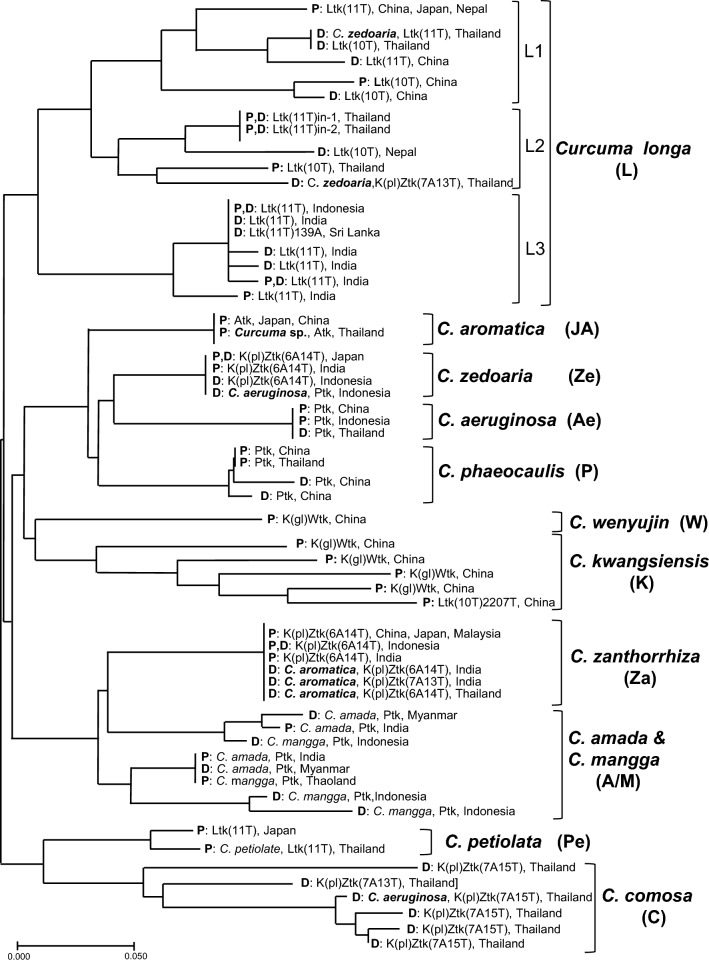
Dendrogram constructed by neighbor-joining method based on similarity of the ILP patterns. The scale under the tree indicates branch length. For each material, plant specimen (**P**) or crude drug sample (**D**), *trn*K sequence type, and production area are shown. When the botanical origin of crude drug samples inferred from the local name was different from the result of molecular analysis, the inferred name was also added

To elucidate specific molecular markers of medicinally used *Curcuma* species in Asia, and to resolve the confusion on the reported botanical origin of crude drugs, molecular analysis based on ILP in *DCS* and *CURS* genes and *trn*K gene sequences was performed using 59 plant specimens and 42 crude drug samples from 13 *Curcuma* species, obtained from Asian countries including China, Japan, Thailand, Indonesia, India, Nepal, Malaysia, Myanmar, and Sri Lanka [28]. The plant specimens, mostly introduced from the former seven countries, were collected from several medicinal plant gardens in Japan. The botanical origins of crude drug samples were inferred from their local names using relevant literature as a reference. Among these, *C. comosa* Roxb. was identified as the likely botanical origin of the Thai crude drug “Wan chak modluk.” In the ILP analysis, the length of the amplified DNA fragments ranged within 213–276 bp in *DCS* intron I region, 274–308 bp in *DCS* intron II region, and 194–256 bp in *CURS* intron region. The ILP patterns of the respective species revealed high intraspecies consistency in *C. aromatica* from Japan and China; *C. zedoaria* from Japan, Indonesia, and India; and *C. phaeocaulis*, *C. aeruginosa* Roxb., *C. wenyujin*, and *C. zanthorrhiza* Roxb.; but showed intraspecies polymorphism in *C. longa*, *C. kwangsiensis*, *C. amada* Roxb., *C. mangga* Valeton et Zijp, and *C. comosa.* The similarity of ILP patterns in the specimens and samples of the respective species led to clear clustering in the neighbor-joining tree, with 11 main groups corresponding to the respective species: groups **L** (*C. longa*), **JA** (*C. aromatica*), **Ze** (*C. zedoaria*), **A**e (*C. aeruginosa*), **P** (*C. phaeocaulis*), **W** (*C. wenyujin*), **K** (*C. kwangsiensis*), **Za** (*C. zanthorrhiza*), **A/M** (*C. amada* or *C. mangga*), **Pe** (*C. petiolata*), and **C** (*C. comosa*) (Fig. 7). Groups **Pe** and **C** formed a clade, separated from the large clade, which was further divided into two subclades. Group **L** formed one subclade and was further divided into three subgroups and this grouping was highly consistent with the geographical origins of the included samples; they were tentatively assigned as China–Japan (**L1**), Thailand (**L2**), and India–Indonesia (**L3**) groups. Another subclade comprising the other species was further divided into two branches: one composed of groups **JA**, **Ze**, **Ae**, **P**, **W**, and **K**; and the other composed of **Za** and **A/M**. Intraspecies polymorphism in group **L** is believed to arise from a combination of geographic variation and the presence of numerous cultivars in *C. longa.* For groups **A/M** and **C**, the polymorphism is attributed to taxonomic confusion and instances of crossbreeding in C. *amada*, *C. mangga*, and *C. comosa*. For the *trn*K sequence, in addition to five typical sequences [Ltk, Ptk, Atk, K(pl)Ztk, and K(gl)Wtk], two new types were found with high similarities to the K(pl)Ztk type, but differing in poly-A and poly-T numbers at positions 205 and 502, respectively. The K(pl)Ztk type of sequence detected in *C. zedoaria* from Japan had six As and 14 Ts at these two sites [renamed K(pl)Ztk(6A14T)], whereas new types had seven As and 15 or 13 Ts—named K(pl)Ztk(7A15T)[LC636648] or K(pl)Ztk(7A13T) [[LC636649], respectively. *Curcuma aeruginosa*, *C. amada*, and *C. mangga* possessed the Ptk type of sequence; *C. petiolata*, the Ltk type; and *C. zanthorrhiza*, the K(pl)Ztk(6A14T) type. The five crude drug samples from Thailand, four “Wan chak modluk” and one “Wan maha mek,” which belonged to group C in ILP pattern (*C. comosa*) were of K(pl)Ztk(7A15T) type. The three Thai samples, one “Wan chak modluk” of group C and two “Kamin oi” of group L2 in ILP pattern, possessed the K(pl)Ztk(7A13T) type of sequence. Considering that group L2 was of *C. longa*, the botanical origins of these “Khamin oi” were determined to be a hybrid between *C. longa* (paternal line) and another species (maternal line) with a K(pl)Ztk(7A13T) type of *trn*K sequence. This species might be *C. comosa*, even though “Khamin oi” was inferred to be *C. zedoaria* based on Thai literature. In a similar situation, although the crude drug “Kasturi manjal” from India and “Wan narn kum” from Thailand were considered to be *C. aromatica* in both countries, the botanical origins of two and one samples obtained were demonstrated as *C. zanthorrhiza* belonging to group **Za** in ILP pattern and with mostly K(pl)Ztk(6A14T) type of *trn*K sequence. Therefore, these crude drugs should be used with caution. Moreover, molecular information showed that *C. aromatica* and *C. zedoaria* cultivated in Japan were closely related to *C. aromatica* from China and Thailand, and *C. zedoaria* from Indonesia and India, respectively. Thus, ILP markers in *DCS* and *CURS* genes combined with *trn*K gene sequences were demonstrated as useful for taxonomic arrangement of Asian *Curcuma* species and standardization of Asian *Curcuma* drugs. For more concise results regarding these difficult questions, however, further study including morphological comparison with the specimens from type locality and molecular investigation on variability of ILP pattern in hybrid plants is needed.

To elucidate the sequence differences in intron regions of the *DCS* and *CURS* genes and to search for specific sequences suitable for identification of *Curcuma* species, six plant specimens from five *Curcuma* species (*C. longa*, *C. zedoaria*, *C. phaeocaulis*, *C. aromatica*, and *C. zanthorrhiza*) that showed distinct ILP patterns were subjected to subcloning coupled with sequencing analysis for the *DCS* intron I and *CURS* intron regions [36]. More than 30 sequences of each region from each specimen were grouped into genes *DCS1*, *DCS2*, or *CURS1–CURS3* and subsequently the sequences of the same genes were compared. Sequences belonging to the same gene showed inter-species similarity, and thus these intron sequences were less informative within each single gene region. The determined sequences from each specimen showed 3–5 kinds of sequence lengths in *DCS* intron I region, and 5–7 kinds of sequence lengths in the *CURS* intron region. The fragment numbers and lengths matched those of the corresponding ILP patterns, effectively clarifying the origin of the ILP pattern in *Curcuma* species.

#### EO composition of rhizomes of Asian *Curcuma* species and related crude drugs analyzed by headspace solid-phase microextraction coupled with gas chromatography–MS (HS-SPME–GC–MS)

The EOs comprising various bioactive compounds have been widely detected in *Curcuma* species. Due to the widespread distribution and misidentification of *Curcuma* species and differences in processing methods, inconsistent reports on major compounds in rhizomes of the same species from different geographical regions are not uncommon. This inconsistency leads to confusion and inaccuracy in compound detection of each species and also hinders comparative study based on EO composition. Then, to characterize EO compositions of *Curcuma* species, as well as to determine the compositional variation among different species, and between plant specimens and their related crude drug samples, 47 plant specimens of 11 *Curcuma* species and 20 crude drug samples identified genetically were analyzed using HS-SPME–GC–MS [37]. Plant specimens of the same species showed similar EO patterns, regardless of geographical source. Based on EO patterns, all specimens and samples were separated into eight main groups: *C. longa* (**L**); *C. phaeocaulis*, *C. aeruginosa*, and *C. zedoaria* (**P-Ae-Ze**); *C. zanthorrhiza* (**Za**); *C. aromatica* and *C. wenyujin* (**JA-W**); *C. kwangsiensis* (**K**); *C. amada* and *C. mangga* (**Am-M**); *C. petiolata* (**Pe**) group; and *C. comosa* (**C**) groups (Thai crude drug samples were used). From EOs of all specimens and samples, 54 compounds (**C1–C54**) were identified (Table 1). The eight groups contained characteristic sesquiterpenes as well as monoterpenes belonging to the bisabolane type (**L**); the elemane- and germacrane-types (**P-Ae-Ze)**; the bisabolane-type (**Za)**; the germacrane-type besides eucalyptol (**C5**) (**JA-W)**; the elemane- and germacrane-types besides camphor (**C10**) (**K)**; the caryophyllane-type besides *β*-pinene (**C2**) and *β*-myrcene (**C3**) (**Am-M)**; the caryophyllane- and germacrane-types besides **C2** and **C5 (Pe**); and the humulane-type besides **C5** (**C**), recognizing two subtypes whether the santalene- and bisabolane types were simultaneously present (type I) or not (type II) (Table 2). Most of the major compounds detected in the plant specimens were also observed in crude drug samples, although a few compounds such as *β*-curcumene (**C26**), turmerone (**C40**), and 4,5-epoxygermacrone (**C52**) degraded due to processing procedures or over time. We supposed **C26** may convert into ar-curcumene (**C29**), and **C52** into curcumenol (**C51**). Two “Khamin oi” samples from Thailand determined genetically to be hybrids between *C. longa* and *C. comosa* with a K(pl)Ztk(7A13T) type of *trn*K sequence, contained major compounds similar to *C. longa* and *C. comosa* type II. Identification of the marker compounds to discriminate each group or each species was achieved after re-discrimination study using the GC–MS data of 47 plant specimens using OPLS-DA. For example, specimens belonging to group **P-Ae-Ze** were used for OPLS-DA, and *C. phaeocaulis*, *C. aeruginosa*, and *C. zedoaria* were separated from each other by curzerenone (**C41**) and curzerene (**C32**); zingiberene (**C23**), ar-curcumene (**C29**), and germacrone (**C43**); and **C5** and **C10**, respectively [37]. By relying on several marker compounds of each species and considering the likelihood of degraded compounds, the identification of Asian *Curcuma* drugs can be achieved to a reasonable degree.

**Table 1:** Essential oil compounds from *Curcuma* specimens and related crude drug samples

	Compounds		Compounds
C1	*α*-Pinene	C28	*β*-Sesquiphellandrene
C2	*β*-Pinene	C29	ar*-*Curcumene
C3	*β*-Myrcene	C30	Germacrene B
C4	*α*-Phellandrene	C31	*p*-Cymen-8-ol
C5	Eucalyptol	C32	Curzerene
C6	*β*-Ocimen	C33	*β*-Caryophyllene oxide
C7	*p*-Cymene	C34	(*E*)-Sesquisabinene hydrate
C8	Terpinolene	C35	Humulene epoxide analog
C9	*δ*-Elemene	C36	(*E*)-Nerolidol
C10	Camphor	C37	*β*-Elemenone
C11	Linalool	C38	Zingiberenol
C12	Santalene	C39	*β*-Cyclodihydrocostunolide
C13	(*E*)-*α*-Bergamotene	C40	Turmerone
C14	*β*-Elemene	C41	Curzerenone
C15	*β*-Caryophyllene	C42	*m*-Camphorene
C16	Guaia-6,9-diene	C43	Germacrone
C17	(*E*)-*β*-Famesene	C44	*β*-Turmerone
C18	*α*-Humulene	C45	ar-Turmerone
C19	(*Z*)-*β*-Farnesene	C46	(6*RS*,7*RS*)-Bisabolone
C20	*γ*-Curcumene	C47	Neocurdione
C21	Germacrene D	C48	Ambrial
C22	*β*-Selinene	C49	Isocurcumenol
C23	Zingiberene	C50	Curdione
C24	*α*-Selinene	C51	Curcumenol
C25	*β*-Bisabolene	C52	4,5-Epoxygermacrone
C26	*β*-Curcumene	C53	Curcumenone
C27	*δ*-Cadinene	C54	Xanthorrhizol

Compounds with relative content more than 1% are shown

**Table 2:** Major compounds in eight groups defined on the basis of essential oil containing patterns

Group	Species included	Sesquiterpenes	Monoterpenes
**L**	*C. longa*	Bisabolane type: C23, C28, C29, C40*, C44, C45	
**P-Ae-Ze**	*C. phaeocaulis*, *C. aeruginosa*	Elemane type: C41, C14; Germacrane type: C52*, C43	
	* C. zedoaria*		
**Za**	*C. zanthorrhiza*	Bisabolane type: C26*, C29, C54	
**JA-W**	*C. aromatica*, *C. wenyujin*	Germacrane type: C47, C52, C43; Carabrane type: C53	C5
**K**	*C. kwangsiensis*	Elemane type: C32, C14; Germacrane type: C21	C10
**Am-M**	*C. amada*, *C. mangga*	Caryophyllane type: C15	C2, C3
**Pe**	*C. petiolata*	Caryophyllane type:C15; Germacrane type: C52, C43	C2, C5
**C**	*C. comosa* (crude drug)	Type I: Humulane type: C35, C18; Santalene type: C12, C13; Bisabolane type: C25 Type II: Humulane type: C35, C18	C5, C31

Name of compounds is shown in Table 1

*In crude drug samples, the compound degraded due to processing procedures or over time. C52 may convert into C51, and C26 into C29

In summary, molecular analyses of the ILP markers in the *DCS* and *CURS* genes and *trn*K gene sequences combined with EO composition analysis were demonstrated to be useful for taxonomic resolution of Asian *Curcuma* species and the standardization of *Curcuma* crude drugs.

#### Application of quality evaluation results on Asian *Curcuma* crude drugs to JP

The medicinal properties of *C. longa* are mainly attributed to its abundant content of curcuminoids including curcumin, demethoxycurcumin, and bisdemethoxycurcumin, reportedly possessing anti-inflammatory, anti-oxidant, and anti-cancer activities [38], as well as anti-metastatic activity of cancer [39]. From Supplement II to JP15 [40], Turmeric, “Ukon” in Japanese (the Latin name, Curcumae Rhizoma was changed to Curcumae Longae Rhizoma in Supplement I to JP17 [41]) is prescribed to contain not less than 1.0% and not more than 5.0% of total curcuminoids using the LC assay described in the JP with the standard solution of curcumin. Moreover, one identification test must be done using the sample solution as directed in the assay to confirm that the peak area of curcumin is larger than that of demethoxycurcumin, and is larger than 0.69 times that of bisdemethoxycurcumin, which means the content of curcumin is higher than those of both other curcuminoids [6]. Before such a standard was determined, we obtained the following HPLC results to quantify each curcuminoid: turmeric samples from Japanese, Chinese, Indian, and Thailand markets contained 0.24–3.64%, 0.56–4.47%, 2.02–4.08%, and 3.28–4.92% of total curcuminoids, respectively, and curcumin occupied 54–72% of total curcuminoids in all samples. Among other *Curcuma* crude drugs, “Khamin oi” samples from Thailand derived from a hybrid between *C. longa* and *C. comosa* with a K(pl)Ztk(7A13T) type of *trn*K sequence contained 1.2–2.0% curcuminoids, in which demethoxycurcumin content exceeded that of curcumin [30]. Therefore, this identification test, as well as quantifying curcuminoids, is essential to avoid curcuminoid-containing crude drugs other than turmeric.

For the other *Curcuma* crude drug, “Gajutsu” in Japanese, although *C. zedoaria* has been prescribed as the botanical origin until Supplement I to JP17 [41], *C. phaeocaulis* and *C. kwangsiensis* were newly added, and the English and Latin names were changed to Curcuma Rhizome and Curcumae Rhizoma from Zedoary and Zedoariae Rhizoma, respectively. As mentioned above, Gajutsu samples derived from *C. kwangsiensis* cultivated in Guangxi Zhuangzu Autonomous Region lacked genetic and chemical stability due to their hybrid origin, whereas those from *C. phaeocaulis* in Sichuan Province and those from *C. zedoaria* in Japan were stable. Since Gajutsu has been used to treat “Oketsu” in Japanese (the syndrome of blood stasis, insufficient blood circulation, sometimes caused by inflammation) [5], the anti-Oketsu effects were examined by two pharmacological studies using genetically identified crude drugs: effects on vasomotion in rat aortic rings, especially on endothelial-dependent relaxation effect of the blood vessel [42]; and anti-inflammatory activity using an arthritis mice model induced by Complete Freund’s Adjuvant injection [43]. In the former study, all methanol extracts of five *Curcuma* drugs exhibited intense nitric oxide (NO)-independent relaxation and all water extracts showed relaxation effects as a sum of the methanol-soluble compounds-induced relaxation and polysaccharides-induced contraction. Only the water extract of *C. zedoaria* showed NO-dependent relaxation and NO-independent relaxation, suggesting the drug derived from *C. zedoaria* has the potential to cure Oketsu through various acting points [42]. In the latter study, the methanol extract of *C. phaeocaulis* significantly inhibited paw swelling in the right hind footpad of mice and serum haptoglobin concentration, whereas that of *C. kwangsiensis* significantly inhibited serum haptoglobin concentration when orally administrated 1 day before the injection. Moreover, for in vitro assay, cyclooxygenase (COX)-2 activity was significantly inhibited by the methanol extract of *C. phaeocaulis*. Therefore, *C. phaeocaulis* could be a useful drug among *Curcuma* species for treating acute inflammation [43]. Subsequently, furanodienone and **C51** were identified as the major active anti-inflammatory constituents of *C. phaeocaulis*, through a new approach for investigation of bioactive constituents in crude drugs using partial least squares regression model and detailed analysis of the regression vector, followed by isolation of these compounds and their COX-2 inhibitory assays. The COX-2 inhibitory activities of furanodienone and **C51** were weaker (IC_50_ 4.6 and 34.5 μM, respectively) than those of the positive control indomethacin; however, selectivity indices (IC_50_ of COX-1/IC_50_ of COX-2) of both compounds (11.4 and 4.6) were higher than that of indomethacin (0.05) [44]. In previous section, franodienone was not mentioned, because this compound was heat-sensitive and could be completely transformed into **C41** in our GC–MS condition [45]. According to our quantitative analysis using the HPLC method, the methanol extract of *C. phaeocaulis* rhizome contained franodienone and **C41** at an average ratio of 2.5:1. Referring to the aforementioned results and considering the distribution amounts, it is evident that *C. phaeocaulis, C. kwangsiensis*, and *C. zedoaria* are the likely botanical origins of Gajutsu, Curcuma Rhizome as recognized in the JP [6].

## Conclusion

To achieve the sustainable use of crude drugs and ensuring their stable quality, comprehensive studies on genetic, chemical, and sometimes pharmacological diversity of Asian medicinal plants including *Paeonia lactiflora*, *Glycyrrhiza uralensis*, *Ephedra* spp., *Saposhnikovia divaricata*, and *Curcuma* spp., as well as their related crude drugs were conducted using several strategies. (1) For peony root, after genetic and chemical diversity analysis of crude drug samples including WPR and RPR in China, the suitable and value-added resources with quality similar to RPR were explored among many horticultural *P. lactiflora* varieties, and two varieties were identified. In addition, an optimized processing method after harvest, which enabled high contents of the main active components in the produced root, was developed to promote cultivation and production of brand peony root. (2) Alternative resources of glycyrrhiza, ephedra herb, and saposhnikovia root and rhizome of JP grade were discovered mostly in eastern Mongolia after field investigation and quality assessment comparing Mongolian plants with Chinese crude drugs. At the same time, suitable specimens and prospective regions for cultivation were proposed. (3) Because of the wide distribution and morphological similarities of *Curcuma* species, classification of some species is debated, which leads to confusion in use of *Curcuma* crude drugs. Molecular analyses of the ILP markers in the *DCS* and *CURS* genes and *trn*K gene sequences combined with the EO composition analysis were demonstrated as useful for standardization of *Curcuma* crude drugs. Molecular and chemical markers to identify botanical origins were elucidated. The aforementioned studies, representing various facets, can be applied to other crude drugs. We anticipate numerous studies focusing on the sustainable use of crude drugs in the future.

### Supplementary Information

Below is the link to the electronic supplementary material.

Supplementary file1 (DOCX 102 KB)
