# Supplementary material for: Comprehensive study on genetic and chemical diversity of Asian medicinal plants, aimed at sustainable use and standardization of traditional crude drugs
Source: J Nat Med. 2023 Dec 22;78(2):267–84. doi: 10.1007/s11418-023-01770-2 (PMC10902101; doi:10.1007/s11418-023-01770-2)
Supplement: Supplementary file 1 — Supplementary file1 (DOCX 102 KB) [file 11418_2023_1770_MOESM1_ESM.docx]

Supplementary Information

**Comprehensive study on genetic and chemical diversity of Asian medicinal plants, aimed at sustainable use and standardization of traditional crude drugs**

Katsuko Komatsu

*Institute of Natural Medicine, University of Toyama, 2630 Sugitani, Toyama 930-0194, Japan*

*Corresponding author*

*E-mail Address*: katsukok@inm.u-toyama.ac.jp (K. Komatsu)

Phone: +81-76-4347648

ORCID

Katsuko Komatsu: 0000-0002-0833-7199

**Table S1　Plant specimens used in the study on genetic and chemical diversity of *Paeonia lactiflora* and peony root**

| Code No. | Plant | W/C^a^ | Collecting location | Voucher No. | Collection Date |
| --- | --- | --- | --- | --- | --- |
| P1 | *Paeonia lactiflora* | W | Duolun, Inner Mongolia, China | Wei S.L., 2007-1 | 2007.01.28 |
| P2 | *P. lactiflora* | W | Arxan, Inner Mongolia, China | Murakami M. MM-1 | 2007.08.11 |
| P3 | *P. lactiflora* | W | Mongolia | 623-63 | 1967 |
| P4 | *P. anomala* | W | Tuluugiyn davas-Hutag-Ondor, Bulgan, Mongolia | Komatsu K. et al., M505 | 2002.07.20 |
| P5 | *P. anomala* | W | Jingiin halzan-Bayannuul, Uvs, Mongolia | Komatsu K. et al., M755-1 | 2002.07.29 |
| P6 | *P. anomala* | W | Mongolia | Komatsu K. et al., M755-2 | 2002.07.29 |
| P7 | *P. veitchii* | W | Ganzi, Sichuan, China | Komatsu K. et al., S-1 | 1996.07.10 |
| P8 | *P. japonica* | C | Toyama, Japan | Murakami M., MM-4 | 2008.12.04 |
| P9 | *P. lactiflora* | C | Xinwo, Zhejiang, China | Komatsu K. et al., CJZ132 | 2009.08.05 |
| PL1 | *P. lactiflora* | W | Arong, Inner Mongolia, China | Komatsu K. et al. HJN67 | 2012.07.20 |
| PL2 | *P. lactiflora* | W | Arong, Inner Mongolia, China | Komatsu K. et al. HJN70 | 2012.07.20 |
| PL3 | *P. lactiflora* | W | Chifeng, Inner Mongolia, China | Komatsu K. et al. HJN252 | 2012.08.04 |
| PL4 | *P. lactiflora* | W | Duolun, Inner Mongolia, China | Komatsu K. et al. HJN295 | 2012.08.05 |

^a^ W: Wild; C: Cultivated

**Table S2 　Crude drug samples used in the study on genetic and chemical diversity of *Paeonia lactiflora* and peony root**

^a^ Underlined number: multiple individuals in the sample were analyzed molecularly.

^b^ Botanical origin of each sample was identified by ITS sequence. WPR-PL indicates WPR-type of *P. lactiflora*, RPR-PL indicates RPR-type of *P. lactiflora*. PV and PJ represent *P. veitchii* and *P. japonica*, respectively.

^c^ The specimen reference number of the Museum of Materia Medica, Institute of Natural Medicine, University of Toyama.

**Table S3　Cultivars of genus *Paeonia* used in the study on genetic and chemical diversity of *Paeonia lactiflora* and peony root**

| Code No. | Cultivar’s Name | Code No. | Cultivar’s Name | Code No. | Cultivar’s Name | Code No. | Cultivar’s Name |
| --- | --- | --- | --- | --- | --- | --- | --- |
| S1* | Haru-no-yosooi | S21* | Rainbow | S41 | Madame Purple | S72* | Takizawa-aka |
| S2* | Kojima-no-kagayaki | S22 | Krinkled white | S42* | Miss Claim | S73* | Peter Brand |
| S3 | Raspberry sundae | S23* | Benishinano | S43 | Lois Kelsey | S74* | Princess Rose |
| S4 | Haru-no-niji | S24 | Rose Glory | S44* | Sunrize | S75 | Lord Campion |
| S5* | Ebisuna-ru | S25* | Satsuki | S45 | Ogon | S76 | Duchesse de Nemours |
| S6 | Momoyama | S26 | Shirayuki | S46* | Shirotae | S77* | Cheddar Surprise |
| S7* | Silver | S27* | Red Comet | S47* | Hyoten | S78* | Edulis Superba |
| S8 | Highlight | S28* | Avalanche | S48* | Hanakago | S79 | Komazawa |
| S9 | Bridal Icing | S29 | Mine-no-yuki | S49* | Kashoku-no-ten | S80** | Oriental Gold |
| S10 | Rosario | S30 | Fujimusume | S50* | Taki-no-yosooi | S81 | Honey Gold |
| S11 | Meigetsu | S31^a^ | Kitasaisho | S51* | Shinano-no-yuki | S83* | Gerry |
| S12 | Sweet Sixteen | S32* | Roosevelt | S52* | Alps | S85* | Kansas |
| S13 | Koga | S33* | Yukuharu | S53 | Richard | S86* | Shinano No. 3 |
| S14* | Haresugata | S34^a^ | Bonten | S54* | Yatoris | S91 | Festiva Supreme |
| S15 | Blue Sapphire | S35* | Festiva Maxima | S58* | Yuubae | S92* | Yamabiko |
| S16* | Shinano-no-haru | S36* | Rinbu | S61* | Martha Reed | S93* | La Tendresse |
| S17* | Miyama-no-yuki | S37 | Primavera | S62* | General MacMahon | S94 | Bridal Shower |
| S18* | Venus | S38 | Flora | S69* | Sarah Bernhardt | S95** | Etched Salmon |
| S19 | Harmony | S39* | White Ivory | S70 | Sebastian Maas | S96 | Sugadaira No. 30 |
| S20* | Red Baron | S40* | Sanadanishiki | S71* | Aratama | S97** | Miss America |
|  |  |  |  |  |  | S98 | Gion |

All cultivars were obtained from Toyama Prefectural Medicinal Plants Center, Toyama, Japan in 2007 and 2009-2011.

^a^ Medicinal cultivars of *P. lactiflora*; others, horticultural cultivars of *P. lactiflora*

* RPR-type of *P. lactiflora*, determined by ITS sequence

** Hybrid between *P. lactiflora* and other *Paeonia* species; S80: *P. lactiflora* x (*P. lutea* x *P. suffruticosa*), S95: *P. officinale* x *P. lactiflora*, S97: unknown

**Table S4　Inhibitory effects of compounds isolated from Red Peony Root sample and the root of “Edulis Superba”**

**against DNP-BSA stimulated β-hexosaminidase release in IgE-sensitized RBL-2H3 cells**

| **Compd.** | **IC_50_ (μM)** | **Compd.** | **IC_50_ (μM)** | **Compd.** | **IC_50_ (μM)** |
| --- | --- | --- | --- | --- | --- |
| P1 | 76.20 ± 5.5^a^ | P11 | 76.68 ± 4.6 | P21 | NA |
| P2 | ― | P12 | 40.34 ± 3.2^c^ | P22 | 78.11 ± 3.1 |
| P3 | NA^b^ | P13 | NA | P23 | 50.12 ± 1.2 |
| P4 | 42.22 ± 1.8 | P14 | NA | P24 | 88.92 ± 2.7 |
| P5 | 75.44 ± 2.5 | P15 | NA | P25 | 48.84 ± 1.4^c^ |
| P6 | NA | P16 | 41.17 ± 3.2 | P26 | 62.50 ± 1.8^c^ |
| P7 | 50.05 ± 3.9 | P17 | NA | P27 | 25.05 ± 4.0^c^ |
| P8 | 68.32 ± 2.7 | P18 | 59.88 ± 3.7 | P28 | 42.55 ± 2.0^c^ |
| P9 | 87.11 ± 1.1 | P19 | 62.00 ± 1.4 | P29 | 60.70 ± 1.1^c^ |
| P10 | 77.51 ± 2.0 | P20 | NA |  |  |

^a^ The results are expressed as the mean±S.D., IC_50_ values were calculated by Probit regression analysis of SPSS software (version 15.0); ^b^ IC_50_ values are more than 100 μM; ^c^ IC_50_ value of compounds isolated from the cultivar, Edulis Superba (ref. 16) and others for compounds from Red Peony Root sample (ref. 15).

**P1**: paeoniflorin, **P2**: paeoniflorin sulfonate, **P3**: 4-*O*-methylpaeoniflorin, **P4**: salicylpaeoniflorin, **P5**: benzoylpaeoniflorin, **P6**: mudanpioside C, **P7**: galloylpaeoniflorin, **P8**: mudanpioside J, **P9**: oxypaeoniflorin, **P10**: benzoyloxypaeoniflorin, **P11**: 6’-*O*-vanillyloxypaeoniflorin, **P12**: mudanpioside E, **P13**: albiflorin, **P14**: 4-*epi*-albiflorin, **P15**: paeonivayin, **P16**: paeoniflorol, **P17**: 4’-hydroxypaeoniflorigenone, **P18**: lactiflorin, **P19**: 1,2,3,4,6-penta-*O*-galloyl-β-d-glucose, **P20**: (+)-catechin, **P21**: paeonol, **P22**: gallic acid, **P23**: methyl gallate, **P24**: benzoic acid, **P25**: paeonolide, **P26**: paeonibenzofuran, **P27**: quercetin, **P28**: quercetin-3-*O*-β-d-glucopyranoside; **P29**: (*2R*)-(-)-naringenin-7-*O*-β-d-glucopyranoside

Fig. S1 Contents of 8 components in the roots of *Paeonia lactiflora* cultivars from Toyama Prefectural Medicinal Plants Center

The cultivars with no less than 2.0% of **P1** are shown. The cultivars’ names are indicated in Table S3.

Quantitative analysis was performed by HPLC method described in ref. 8. Average contents of **P1** and **P13** are shown in upper part and those of **P19**-**P24** in lower part (n=2-4).
